# Oxidative stress and apoptosis in a pig model of brain death (BD) and living donation (LD)

**DOI:** 10.1186/1479-5876-11-244

**Published:** 2013-10-02

**Authors:** Philipp Stiegler, Michael Sereinigg, Andreas Puntschart, Andrea Bradatsch, Thomas Seifert-Held, Iris Wiederstein-Grasser, Bettina Leber, Elke Stadelmeyer, Nadia Dandachi, Siglinde Zelzer, Florian Iberer, Vanessa Stadlbauer

**Affiliations:** 1 Division of Surgery, Department of Transplantation Surgery, Medical University, Auenbruggerplatz 29, Graz 8036, Austria; 2 Department of General Surgery, Medical University, Graz, Austria; 3 Department of Neurology, Medical University, Graz, Austria; 4 Division of Biomedical Research and Section for Surgical Research, Medical University, Graz, Austria; 5 Department of Internal Medicine, Division of Gastroenterology and Hepatology, Medical University, Graz, Austria; 6 Department of Internal Medicine, Division of Oncology, Medical University, Graz, Austria; 7 Clinical Institute of Medical and Chemical Laboratory Diagnostics, Medical University, Graz, Austria

**Keywords:** Organ donation, Brain death, Living donation, Oxidative stress, Apoptosis

## Abstract

**Background:**

As organ shortage is increasing, the acceptance of marginal donors increases, which might result in poor organ function and patient survival. Mostly, organ damage is caused during brain death (BD), cold ischemic time (CIT) or after reperfusion due to oxidative stress or the induction of apoptosis. The aim of this study was to study a panel of genes involved in oxidative stress and apoptosis and compare these findings with immunohistochemistry from a BD and living donation (LD) pig model and after cold ischemia time (CIT).

**Methods:**

BD was induced in pigs; after 12 h organ retrieval was performed; heart, liver and kidney tissue specimens were collected in the BD (n = 6) and in a LD model (n = 6). PCR analysis for NFKB1, GSS, SOD2, PPAR-alpha, OXSR1, BAX, BCL2L1, and HSP 70.2 was performed and immunohistochemistry used to show apoptosis and nitrosative stress induced cell damage.

**Results:**

In heart tissue of BD BAX, BCL2L1 and HSP 70.2 increased significantly after CIT. Only SOD2 was over-expressed after CIT in BD liver tissue. In kidney tissue, BCL2L1, NFKB, OXSR1, SOD2 and HSP 70.2 expression was significantly elevated in LD. Immunohistochemistry showed a significant increase in activated Caspase 3 and nitrotyrosine positive cells after CIT in BD in liver and in kidney tissue but not in heart tissue.

**Conclusion:**

The up-regulation of protective and apoptotic genes seems to be divergent in the different organs in the BD and LD setting; however, immunohistochemistry revealed more apoptotic and nitrotyrosine positive cells in the BD setting in liver and kidney tissue whereas in heart tissue both BD and LD showed an increase.

## Background

Whole organ transplantation still remains the therapy of choice for several end-stage organ failures. As the demand for organs is increasing steadily, donor selection criteria are expanded and therefore strategies to improve the quality of such marginal donors are needed. It is known that the clinical outcome in kidney transplantation from living donors (LD) is superior to that for transplantation from brain dead (BD) donors [1]. However, most organs are transplanted from BD and, therefore, a focus on characterization of pathophysiological pathways that cause organ damage is essential in order to be able to ameliorate transplantation outcome. BD, due to massive catecholamine release, is followed by significant hemodynamic disturbances and ischemia that occurs before organ retrieval has begun and leads to the induction of oxidative stress [1,2]. Oxidative stress has been implicated in development of complications after organ transplantation emphasizing on ischemia-reperfusion injury (IRI), delayed graft function (DGF) [3] and primary allograft dysfunction which remains a serious problem in organ transplantation [4]. The occurrence of oxidative stress as well as apoptosis and nitrosative stress induced cell damage in LD and BD is therefore of interest. As there exist a lot of small animal experiments in this setting [5,6], but large animal studies are rare, we decided to analyzed apoptosis related genes (NFKB [7], BAX – BCL2 associated X protein [8]; BCL2L1 [9]) as well as oxidative stress related genes (SOD2, GSS, PPARalpha, OXSR1, GPX3, HSP 70.2) in a pig model of LD and BD organ donation. NFKB is known to play a key role in initiation of inflammation and rejection as well as occurrence of apoptosis in organ transplantation [7,10]. Taking in consideration BCL2L1 a product of an anti-apoptotic gene as well as BAX, a pro-apoptotic gene, the susceptibility of cells toward apoptotic stimuli can be defined [8,9,11]. Superoxide dismutase (SOD) encodes for an enzyme that converts superoxide radicals to hydrogen peroxide [12], gluthatione synthetase (GSS) encodes for a key enzyme in prevention of local oxidative stress [13,14], the peroxisome proliferators-activated receptor-alpha (PPARalpha) gene plays a role in energy metabolism and might suppress induction of apoptosis [15], the gene for oxidative stress responsive 1 (OXSR1) controls cell proliferation and cell death by apoptosis [16], gluthatione peroxidase 3 (GPX3) encodes for a cellular anti-oxidant system that protects cells against oxidative stress [17,18] and heat shock protein 70.2 gene expression (HSP 70.2) takes part in processes of protection and repair of stress-induced protein damage [19,20] in the different tissues. The aim of this study was to study a panel of genes involved in oxidative stress and apoptosis and correlate gene expression with immunohistochemical findings of apoptosis and nitrosative stress and serum markers of oxidative stress before and after harvesting of organs from a BD and LD pig model and after cold ischemia time (CIT) in order to have the potential possibility to find novel strategies for donor pre-conditioning and amelioration in further experiments.

## Methods

### BD induction and organ/tissue harvest

12 animals (suus scrofa domestica; 35 ± 3.2kg) from the same origin were used for these experiments. All animal procedures were carried out in accordance with the Austrian Animal Law (66.010/46-II/10b/2009 and 66.010/75-C/GT/2007/C/GT according to BGBl. Nr.501/1989, i.d.F. BGBl. 1 Nr. 169/1999) and experiments were performed in accordance with the NIH guide for the care and use of laboratory animals [21]. Organ procurement from BD (n = 6) and LD animals (n = 6) was performed according to the local standards [22] and the EUROTRANSPLANT guidelines [23]. For BD a slow BD induction model was used as described elsewhere; briefly, after drilling a burr hole in the right frontoparietal region of the skull, a Foley catheter was inserted into the subdural space. The balloon of the catheter was inflated with saline solution at a rate of 3 ml every 30 seconds to a total volume of 24 ml. Animals were kept at a mean arterial pressure of 50 mmHg and animals were stabilized for 10 hours according to local donor management guidelines [22,24]. 60 min after total inflation of the balloon, anaesthesia was discontinued and after a period of another 60 min, BD diagnostic was performed including testing of brain stem reflexes and painful stimuli by a neurologist, followed by apnoea testing and a 30 min 8 - channel EEG exactly as described elsewhere [25]. Only in pigs with an isoelectrical EEG as well as CO_2_ levels above 60 mmHg during the apnoea testing, BD was confirmed. Then, BD pigs were managed according to the current recommendations to manage a multi organ donor. Immediately prior to organ perfusion, immediately after organ perfusion and after defined CIT tissue samples were taken and put either on paraffin for immunohistochemistry or on liquid nitrogen for PCR analysis. CIT was 4 hours for heart tissue, 6 hours for liver and 15 hours for kidney tissue. These time-points were chosen because they represent average acceptable CIT duration for the respective organs.

### Immunohistochemistry and histology

Prior to perfusion, after perfusion and after CIT, tissue samples of the different organs were fixed in formalin and embedded in paraffin and sections of 3 μm were cut with a microtome and stained with hematoxylin and eosin according to standard procedures.

For immunohistochemistry, antibodies against activated human/mouse/pig Caspase 3 (Affinity-Purified Rabbit Anti- Active Antibody, R&D Systems, Minneapolis, MN, USA; dilution 1:50) were applied to deparaffinised and rehydrated sections after antigen retrieval by microwaving for 40 minutes at 160 W in 0.01 M citrate buffer, pH 6.0. Binding of primary antibodies was detected with the iVIEW DAB Detection Kit on Ventana ES automatic stainer (Ventana Medical Systems, Tuscon, Arizona, USA). For negative control, primary antibodies were omitted or replaced by isotype matched immunoglobulins.

For the detection of nitrosative stress an anti-nitrotyrosine antibody (rabbit IgG fraction, Invitrogen, Eugene, Oregon, USA; dilution 1:200) was applied to deparaffinised and rehydrated sections after antigen retrieval using CC1 reagent (Ventana Medical Systems, Tucson, Arizona, USA) for 60 min. After 32 min of incubation with the primary antibody, blocking and detection was performed using the ultraView Universal DAB Detection Kit (Ventana Medical Systems, Tucson, Arizona, USA) on a BenchMark XT automated tissue staining system (Ventana Medical Systems, Tucson, Arizona, USA) according to validated protocols. Hematoxyline served as counterstain for nitrotyrosines.

For the quantification of activated Caspase 3 positive cells as well as nitrosative stress damaged cells, positive cells were evaluated by three independent investigators semi-quantitatively and statistical analysis was performed. Photodocumentation was performed using a Leica^®^ DC 300 or Leica^®^ DC 350 camera (Leica Camera AG, Solms, Germany) and a 40 mm objective (Scan Magnification 40 mm objective, Carl Zeiss GmbH, Vienna, Austria).

### Biochemistry

Full blood count, electrolytes, renal and liver function tests were immediately analysed in the central laboratory continuously prior to BD induction during BD and immediately prior to organ donation. Blood samples were stored at −70°C for analysis of malondialdehyde (MDA) as well as myeloperoxidase (MPO) levels as previously described [26-28].

### RNA Isolation and reverse transcription

Tissue samples were snap-frozen and stored in liquid nitrogen until nucleic acid extraction. Between 50 mg to 100 mg tissue (liver, heart, and kidney) were homogenized by either cutting the tissue into sections with a cryomicrotome and disrupting it in 1 ml TRIzol reagent (Invitrogen, Carlsbad, CA, US) by passing the suspension through a 23 gauge needle or by homogenizing the tissue in 1 ml TRIzol reagent using a MagNA Lyser (Roche Diagnostics GmbH, Mannheim, Germany). Isolation of RNA was done according to the protocol provided by the manufacturer with minor modifications. In brief, disrupted tissue was incubated for 15 minutes at room temperature and treated with 100 μl 1-Bromo-3-chloropropane (Sigma, St. Louis, MO, US) per ml TRIzol reagent. RNA was precipitated in 500 μl isopropyl alcohol (Sigma, St. Louis, MO, US) and the pellet was dissolved in RNase-free water (usb, Cleveland, OH, US). RNA was quantified spectro-photomectrically on a Biophotometer (Eppendorf, Hamburg, Germany) and stored until use at −80°C. One μg RNA was used for reverse transcription (QuantiTect Reverse Transcription Kit; Qiagen, Hilden, Germany) according to the protocol provided by the manufacturer including a step for the elimination of genomic DNA. The final volume of the reaction was 20 μl.

### Primer

Primers were designed based on porcine sequences available online (ENTREZ GENE) with Primer3 software and purchased from Eurofins MWG Operon (Ebersberg, Germany). For the endogenous reference gene HPRT primers were adopted from Nygard et al. [29].

This reference gene was chosen, because it proved high stability across different tissues [29] and is suitable for the quantification of low abundant transcripts. Whenever possible the primers were designed to span exon/intron boundaries to avoid amplification of residual genomic DNA. The specificity of the primers was first evaluated with in-silico analysis. PCR products showed a single melting peak in the melting analysis and a single band on an agarose gel (2.5% agarose in 1-fold TBE running buffer). Sequence specificity was verified by sequence analysis (3730 DNA Analyzer; Applied Biosystems, Foster City, CA, US). Sequences of primers are summarized in Table 1.

**Table 1:** Genes used for quantification and primer information

**Acc. number**	**Forward primer (5' – 3')**	**Reverse primer (5' – 3')**	**Product length**
**HPRT1 (Hypoxanthine phosphoribosyltransferase)**			
NM_001032376	GGACTTGAATCATGTTTGTG	CAGATGTTTCCAAACTCAAC	91 bp
**BAX (BCL2-associated X protein)**			
XM_003127290	GCTGACGGCAACTTCAACTG	CCGATCTCGAAGGAAGTCCA	141 bp
**BCL2L1 (BCL2-like 1)**			
NM_214285.1	TGAGTCGGATCGCAACTTGG	ATCGGTTGAAGCGTTCCTGG	150 bp
**GPX3 (Glutathione peroxidase 3)**			
NM_001115155.1	GAGACAACTCGGAGATTCTG	GGAACGTGTAGAACTTCTGC	126 bp
**GSS (Glutathione synthetase)**			
NM_001244625.1	AAGAAGCTGCCAAGATCCTC	ATTCTCTATGGCACGCTGGT	155 bp
**NFKB1 (Nuclear factor of kappa light polypeptide gene enhancer in b-cells)**			
NM_001048232.1	GAGGTGCATCTGACGTATTC	CACATCTCCTGTCACTGCAT	138 bp
**OXSR1 (Oxidative-stress responsive 1)**			
NM_214342.1	CCGAAGTTATGGAACAGGTC	GATCATTCTGCAGTGTCAGC	147 bp
**PPARA (Peroxisome proliferators-activated receptor- alpha)**			
NM_001044526.1	TGAAGTTCAATGCGCTGGAG	TTGAGCACATGCACGATACC	139 bp
**HSP70.2 (Heat shock protein 70.2)**			
NM_213766.1	AGGTGCAGGTGAGCTACAAG	CTGCGAGTCGTTGAAGTAGG	158 bp
**SOD2 (Superoxide dismutase 2, mitochondrial)**			
NM_214127	CCTACGTGAACAACCTGAAC	GATACAGCGGTCAACTTCTC	247 bp

The efficiency of the primers was determined in a range from 0.4 to 50 ng initial RNA using PCR cycling conditions as described below. Primers showed similar efficiencies (ranging from 1.8 to 2.0) on templates from the three different tissue types and the two storage solutions.

### Real time PCR

Real time PCR amplification and melting analysis were performed using a LightCycler 480 System (Roche Diagnostics GmbH, Mannheim, Germany). cDNA corresponding to an equivalent of 25 ng RNA was added to a reaction mix containing LightCycler 480 SYBR Green I Master (Roche Diagnostics GmbH, Mannheim, Germany) and 0.2 μM of each primer giving a final reaction volume of 20 μl. The PCR reaction mixture was subjected to an initial denaturation at 95°C for 10 seconds, followed by 45 cycles of denaturation at 95°C for 10 seconds, annealing at 58°C at 20 seconds and elongation at 72°C for 30 seconds. Melting analysis started with an initial denaturation at 95°C for 5 seconds followed by an increase in temperature from 65°C to 97°C with 10 acquisitions per degree.

### Data analysis real time PCR

Gene expression was determined using the efficiency method for relative quantification embedded in the LightCycler Software (LCS480 1.5.0.39). Inter-assay variance was calculated using the coefficient of variation (CV) of the mean Cq value of an internal control and was less than 1.8%. cDNA used as template for the internal control was generated from RNA isolated from peripheral blood mononuclear cells (MNC) derived from pigs. Internal control reactions were included with each run and amplified using the HPRT primers. All PCR reactions were done in duplicates.

### Data analysis and statistics

All PCR experiments were carried out as duplicates. Results were normalized to the housekeeping gene (HPRT1) and fold change was calculated as ratio of the target gene expression in the experimental groups (after perfusion or after CIT) to the control group (pre perfusion). Furthermore, to be able to show differences in gene expression between LD and BD, the fold change was also calculated as ration of the target gene expression in the BD group to the LD group. For quantification of immunohistochemistry three blinded, independent persons analysed the slides in a semi-quantitative manner (negative, slightly positive, positive, strongly positive) and the results were transferred to a score form 0–3. The mean of their observations was used for statistical analysis. For comparison of two groups t-test or Mann–Whitney test were used as appropriate, whilst the ANOVA test with Bonferroni´s multicomparison post hoc analysis was used for comparison of more than two data sets. F was >1 for all ANOVA tests. SPSS version 13 and GraphPrism 4 software was used for statistical analysis. A p < 0.05 was considered to be significant.

## Results

### PCR and immunohistochemistry

#### Heart tissue

**Figure 1:**
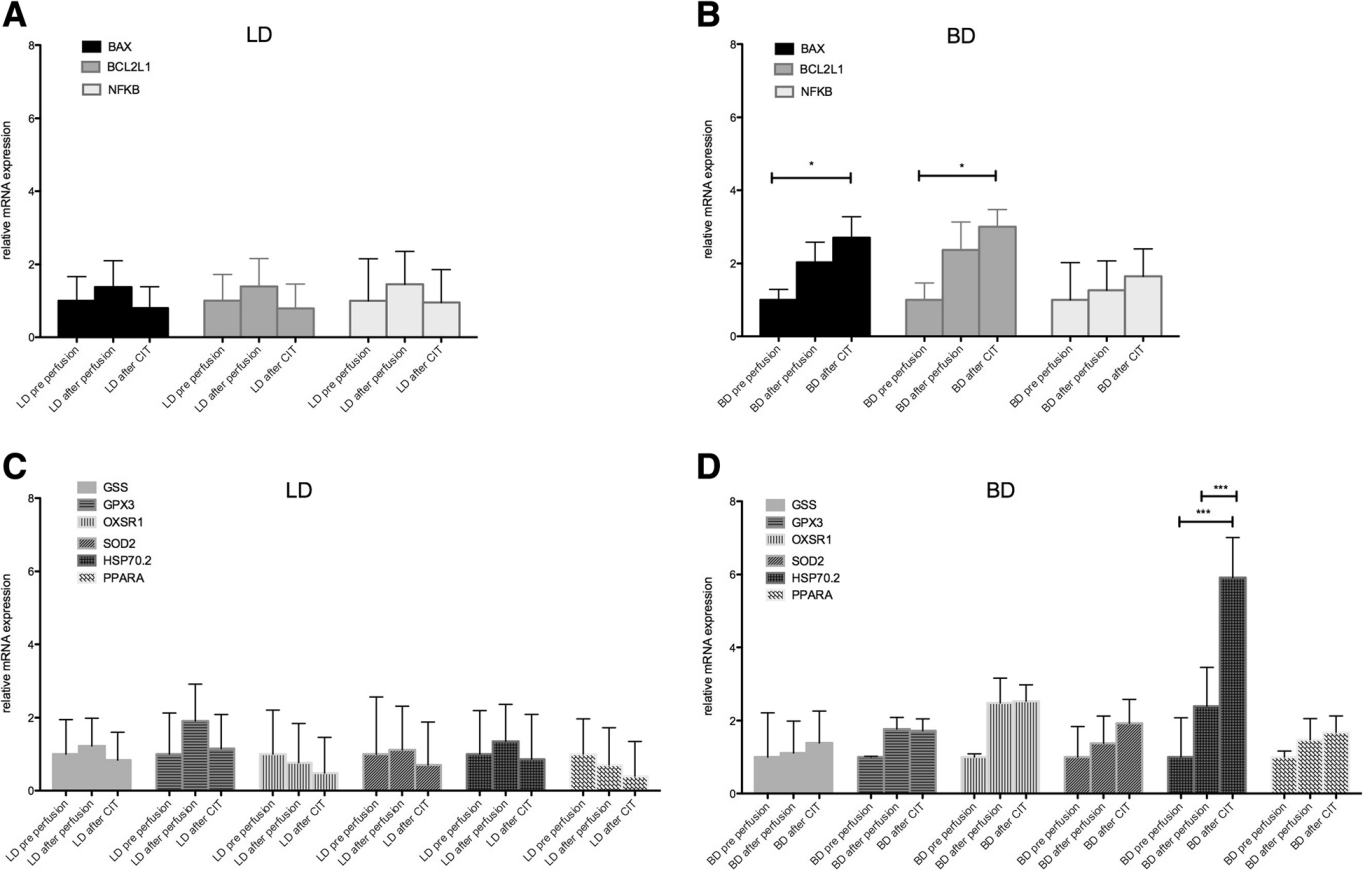
**Apoptosis and oxidative stress related gene expression in pig heart tissue. A)** LD heart apoptosis genes, **B)** BD heart apoptosis genes, **C)** LD heart oxidative stress genes, **D)** BD heart oxidative stress genes *p < 0.05, ** p < 0.01, ***p < 0.001; BD: Brain death; LD: Living donation; CIT: cold ischemic time.

**Figure 2:**
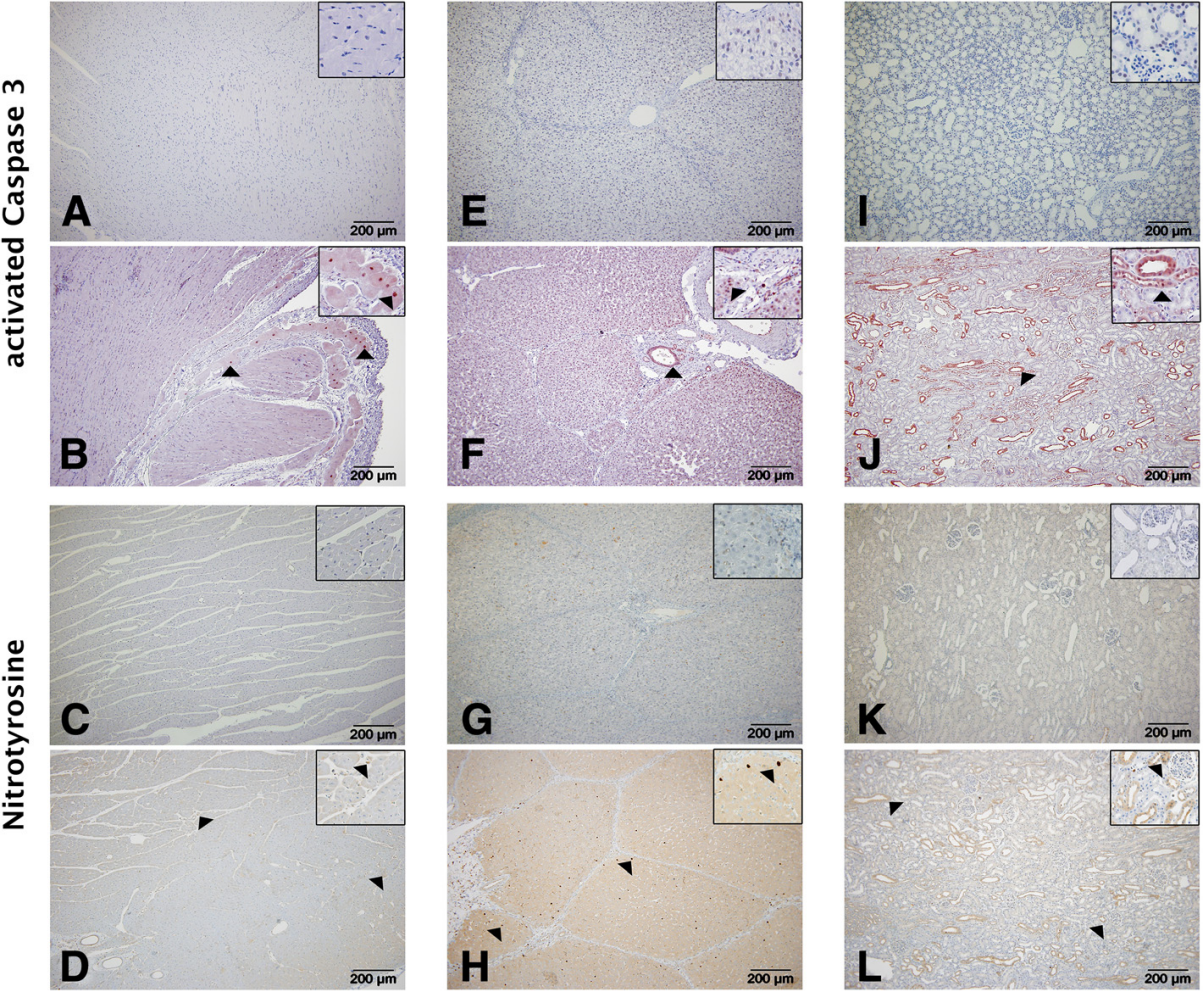
**For immunohistochemistry, an antibody against activated human/mouse Caspase 3 (dilution 1:50) was used for the detection of apoptosis in the different tissues at different time-points.** Heart: **A**, **B**: Representative stainings for heart tissue of BD prior to perfusion **(A)** and after 4 h of CIT **(B)** against activated Caspase 3. **C**, **D**: Representative stainings of nitrotyrosine positive cells in BD hearts prior to perfusion **(C)** and after 6 h of CIT **(D)**. Nitrotyrosine positive cells were more common in BD hearts after CIT, at all other time points no difference between LD and BD was found. Liver: **E**, **F**: Representative stainings of liver tissue of BD prior to perfusion **(E)** and after 6 h of CIT **(F)** against activated Caspase 3. **G**, **H**: Representative stainings for nitrotyrosine positive cells of BD liver tissue prior to perfusion **(G)** and after 6 h of CIT **(H)**. Kidney: **I**, **J**: Representative stainings for kidney tissue against activated Caspase 3 prior to perfusion **(I)** and after 15 h of CIT **(J)**. In kidney tissue, the number of activated Caspase 3 positive cells was significantly higher in both groups after 15 h CIT compared to prior to perfusion and was higher in BD donor organs compared to LD organs after CIT. Especially tubular tissue seemed to be more susceptible to occurrence of apoptosis after CIT. **K**, **L**: Staining for nitrotyrosine positive cells in BD kidney tissue prior to perfusion **(K)** and after 15 h of CIT **(L)**. Tubular cells seemed to be more affected by nitrosative stress in BD kidney tissue after CIT. 100 x magnification. The inserts show areas of interest 200 x magnification. Arrow heads: examples of activated Caspase 3 positive cells **(A**, **B**, **E**, **F**, **I**, **J)** and nitrotyrosine positive cells **(C**, **D**, **G**, **H**, **K**, **L)**; CIT: Cold ischemic time.

In LD heart tissue no change in expression of apoptosis genes (BAX, BCL2L1, NFKB) and oxidative stress genes (GSS, GPX3, OXSR1, SOD2, HSP70.2, PPARA) after perfusion or after CIT as compared to pre perfusion was detected. In BD animals BAX, BCL2L1 and HSP 70.2 expression increased after CIT compared to pre perfusion (2.7 fold, p < 0.05, 3 fold, p < 0.05 and 5.9 fold, p < 0.001 respectively). HSP70.2 expression was also significantly higher after CIT compared to after perfusion (p < 0.001) (Figure 1). The number of activated Caspase 3 positive cells and nitrotyrosine positive cells increased significantly after perfusion and after 4 h of CIT in heart tissue of both BD and LD donor organs compared to the samples taken prior to perfusion. Nitrotyrosine positive cells were more common in BD hearts after CIT, but not at other time-points. Representative stainings are shown in Figure 2 and statistical analysis compiled in Table 2.

**Table 2:** Compiles the results of immunohistochemical evaluation for activated caspase-3 positivity as well as nitrotyrosine positivity for the different organs at the different time-points

***Organ*/IHC**	**LD prior to perfusion**	**LD after perfusion**	**LD after CIT**	**BD prior to perfusion**	**BD after perfusion**	**BD after CIT**
***Heart:***						
**Caspase-3**	0	0	2 ± 0.2 ***	0	1 ± 0.2	2 ± 0.1 ^+++, ###^
**Nitrotyrosine**	0	1 ± 0.3	2 ± 0.2 ***^, ##^	0	1 ± 0.1	3^+++,^ °°°^,§^
***Liver:***						
**Caspase-3**	0	0	1 ± 0.2 *	1 ± 0.1 **	1 ± 0.2	2 ± 0.2 ^+++,^ °°°^,^^§§§^
**Nitrotyrosine**	0	0	1 ± 0.2 ***^, ##^	2 ± 0.6 ***	2 ± 0.3	3 ^++,^ °°^, §§§^
***Kidney:***						
**Caspase-3**	0	0	1 ± 0.1 *	1 ± 0.2	1 ± 0.2	3 ± 0.1 ^+++,^ °°°^, §§§^
**Nitrotyrosine**	0	0	2 ± 0.2 ^***, ###^	1 ± 0.2 **	1 ± 0.2	2 ± 0.1 ^++^

For quantification of immunohistochemistry three blinded, independent persons analysed the slides in a semi-quantitative manner (negative, slightly positive, positive, strongly positive) and the results were transferred to a score form 0–3. The mean of the observations was used for statistical analysis. BD: Brain death; CIT: cold ischemic time; IHC: Immunohistochemistry; LD: Living donation; * p < 0.05; ** p < 0.01; *** p < 0.001 as compared to LD prior to perfusion. ++ p < 0.01; +++ p < 0.001 as compared to BD prior to perfusion. °° p < 0.01; °°° p < 0.001 as compared to BD after perfusion. ## p < 0.01; ### p < 0.001 as compared to LD after perfusion. § p < 0.01; §§§ p < 0.001 as compared to LD after CIT. CIT was 4 hours for heart tissue, 6 hours for liver tissue as well as 15 hours for kidney tissue.

#### Liver tissue

**Figure 3:**
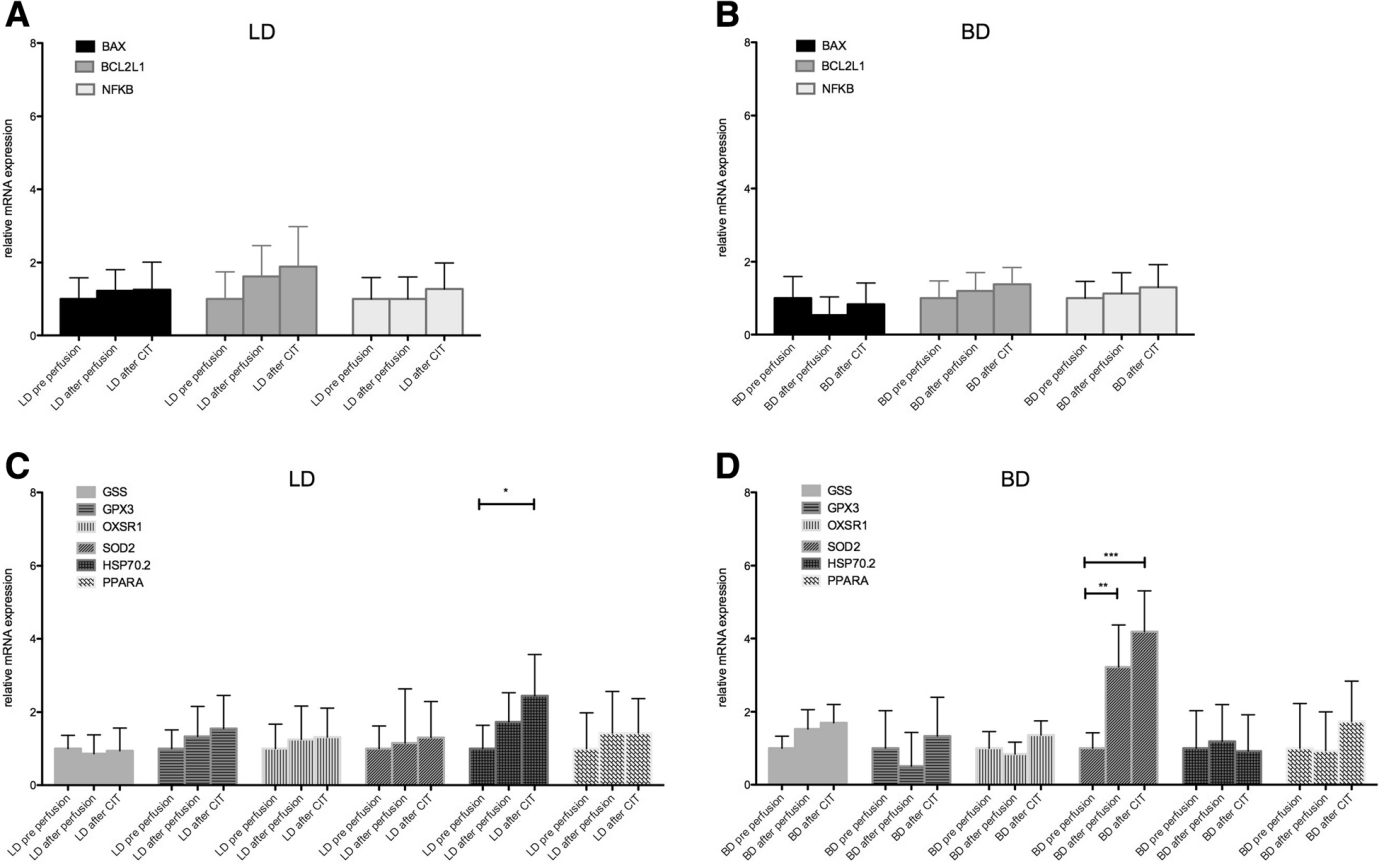
**Apoptosis and oxidative stress related gene expression in pig liver tissue. A)** LD liver apoptosis genes, **B)** BD liver apoptosis genes, **C)** LD liver oxidative stress genes, **D)** BD liver oxidative stress genes *p < 0.05, ** p < 0.01, ***p < 0.001; BD: Brain death; LD: Living donation; CIT: cold ischemic time.

In LD and BD liver tissue no changes in expression levels of apoptosis genes (BAX, BCL2L1, NFKB) could be detected. From the panel of oxidative stress genes, HSP 70.2 was 2.4 fold (p < 0.05) overexpressed after CIT in LD livers, whereas SOD2 was overexpressed after perfusion and after CIT (3.2, p < 0.01 and 4.2 fold, p < 0.001 respectively) in BD livers. (Figure 3) GPX3 was 4.3 fold (p < 0.01) overexpressed after perfusion in LD livers compared to BD livers indicating cell protection (data not shown).

A significant increase in nitrotyrosin positive cells could be observed in both BD and LD livers after perfusion and after 6h of CIT. Caspase 3 positive and nitrotyrosine positive cells were significantly elevated after 6h of CIT in BD organs; activated Caspase 3 and nitrotyrosine positive cells were significantly more common in BD as compared to LD livers prior to perfusion and after perfusion (only nitrotyrosine). Representative stainings are shown in Figure 2 and statistical analysis is compiled in Table 2.

#### Kidney tissue

**Figure 4:**
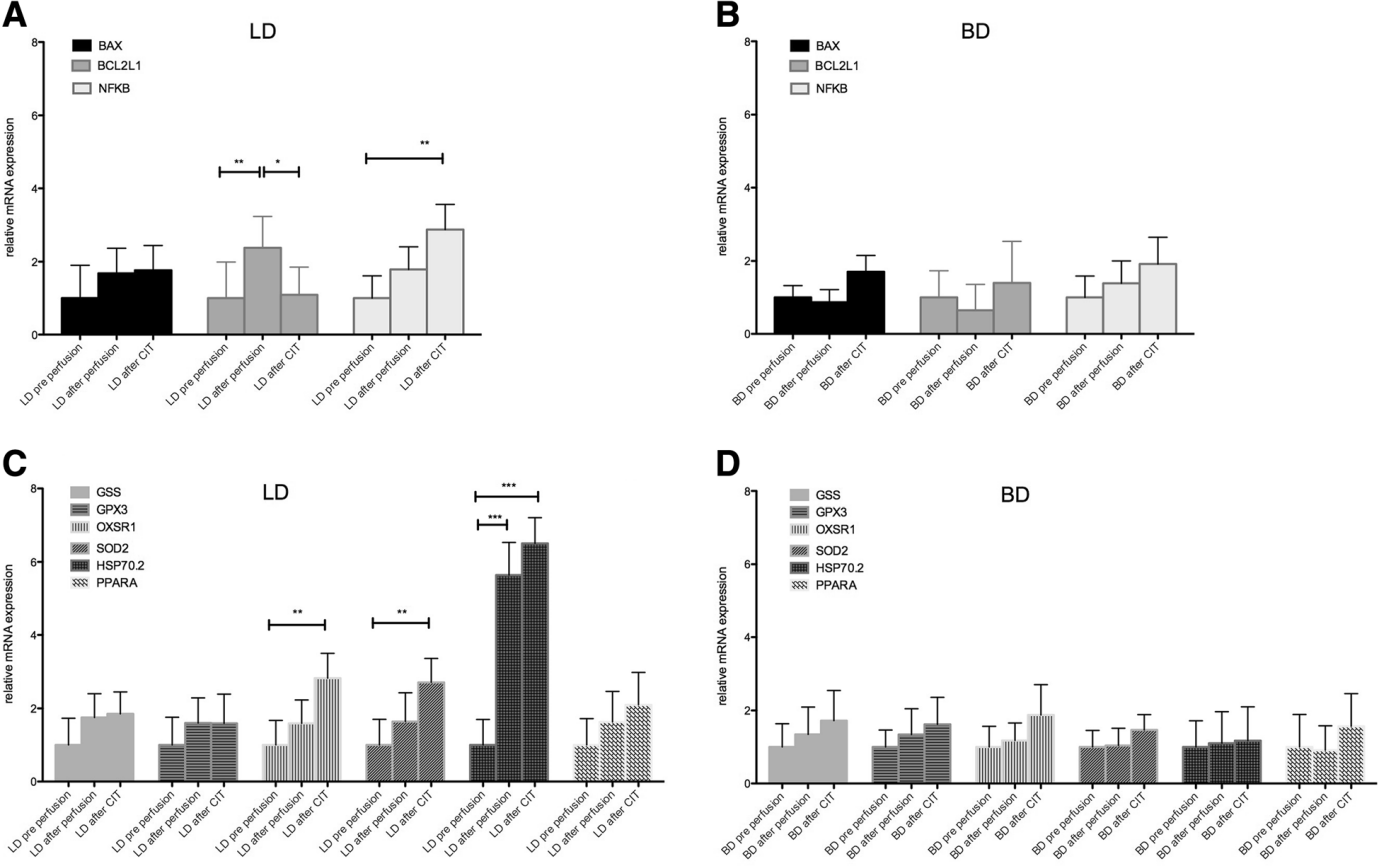
**Apoptosis and oxidative stress related gene expression in pig kidney tissue. A)** LD kidney apoptosis genes, **B)** BD kidney apoptosis genes, **C)** LD kidney oxidative stress genes, **D)** BD kidney oxidative stress genes *p < 0.05, ** p < 0.01, ***p < 0.001 BD: Brain death; LD: Living donation; CIT: cold ischemic time.

In BD kidney tissue no changes in apoptosis and oxidative stress gene expression were observed after perfusion or after CIT compared to pre perfusion. In LD donor kidneys BCL2L1 was 2.4 fold (p < 0.01) over-expressed after perfusion but expression was unchanged when comparing the expression after CIT to pre perfusion. NFKB expression was 2.9 fold (p < 0.01) over-expressed after CIT. OXSR1 (2.8 fold, p < 0.01), SOD2 (2.7 fold, p < 0.01) expression was significantly higher after CIT in LD kidneys and HSP 70.2 expression was significantly higher after perfusion (5.6 fold, p < 0.001) and after CIT (6.5 fold, p < 0.001) (Figure 4). BCL2L1 was 2.4 fold (p < 0.01) over-expressed in LD kidneys after perfusion compared to BD kidneys (data not shown.).

In kidney tissue, the number of activated Caspase 3 positive cells was significantly higher in both groups after 15 h CIT compared to prior to perfusion and was higher in BD donor organs compared to LD organs after CIT. Nitrotyrosine positivity was already significantly higher in BD as compared to LD prior and after perfusion but no difference was observed after CIT. There was also a significant increase in nitrosative stress affected cells in kidney tissue over time in both groups. Representative stainings are shown in Figure 2 and statistical analysis is compiled in Table 2.

### Laboratory parameters

Baseline laboratory findings in LD and BD animals were not different, BD animals showed a significant increase in white blood cells, creatinine, urea, sodium, ALT and GGT 10 hours after induction of brain death. There were no statistically significant differences in MPO and MDA levels immediately prior to organ retrieval between LD and BD (Table 3).

**Table 3:** Laboratory findings in LD and BD pigs LD: Living donation, BD: Brain death

	**LD prior to perfusion**	**Prior to induction of BD**	**BD prior to perfusion**
Red blood cells (10E6μL)	4.3 ± 0.1	4.6 ± 0.2	4.9 ± 0.4
White blood cells (10E3/μL)	14.0 ± 1.3	10.8 ± 1.3	17.4 ± 1.0**
Hemoglobin (g/dL)	7.1 ± 0.2	6.4 ± 0.4	6.9 ± 0.5
Hematocrit (%)	23.9 ± 0.8	27.0 ± 3.0	28.2 ± 2.0
Creatinine (mg/dL)	1.2 ± 0.1	0.7 ± 0.1	1.4 ± 0.1***
Urea (mg/dL)	23.5 ± 2.6	19.8 ± 2.4	29.2 ± 2.5*
Sodium (mmol/L)	139.3 ± 0.7	137.2 ± 0.9	143.7 ± 0.8***
Potassium (mmol/L)	3.7 ± 0.1	4.2 ± 0.2	4.2 ± 0.2
AST (U/L)	34.1 ± 5.3	29.5 ± 3.3	36.2 ± 3.8
ALT (U/L)	34.8 ± 2.9	28.2 ± 3.1	49.5 ± 2.5***
GGT (U/L)	41.9 ± 4.6	37.7 ± 5.4	63.0 ± 1.2**
MDA (μmol/L)	10.2 ± 5.1	-	3.9 ± 0.9
MPO (ng/mL)	4.0 ± 1.5	-	2.9 ± 1.4

ALT: alanine-aminotransferase. AST: aspartate-aminotransferase, gGTgamma-glutamyl transferase. MDA: malondialdehyde. MPO: myeloperoxidase. As there was no statistically significant difference in between LD and BD immediately prior to perfusion, MDA and MPO measurements were not performed prior to BD induction. *p < 0.05, **p < 0.01, ***p < 0.001 compared to before induction of BD.

## Discussion

Since donor organs are a limited resource, organs of different quality have to be used. It is important to understand factors that might impact negatively on organ and patient survival. Oxidative stress as well as occurrence of apoptosis and nitrosative stress induced cell damage in LD and BD might be of interest in order to find new strategies to improve transplantation outcome and patient long-term survival [30-32]. Most of recent studies [5] focus mainly on pro- and anti-apoptotic gene expression during BD not taking in consideration that oxidative as well as nitrosative stress may play a major role in organ damage during BD. Therefore, the aim of this study was to evaluate a panel of genes encoding for protective mechanisms against oxidative stress induced cell damage as well as pro- and anti-apoptotic genes in BD and LD at different time points and to document their influence on organ quality in terms of occurrence of apoptosis mainly after CIT.

The significant pathophysiological alterations caused by BD in a potential organ to be transplanted result in inflammation and injury, which might affect graft function [9,30]. Therefore, a comprehensive understanding of this process is mandatory to improve intervention strategies [33]. After transplantation, IRI is clinically important because it can cause acute organ failure and high patient mortality [34,35]. ROS and proinflammatory cytokines which occur during BD as well as CIT play a key role in the pathophysiology of IRI [35]. In order to successfully target ROS, it is necessary to consider the specific ROS involved, the sources generating ROS in what particular location, at what time in the pathogenesis, and how much oxidative stress is generated. Additionally it is crucial to understand the mechanisms by that ROS are actually causing cell death and organ injury [36]. Under stress conditions such as BD, CIT as well as IRI, mitochondrial dysfunction may occur what impacts on detoxifying ROS in mitochondria or help repair minor changes [36,37]. Different endogenous genes or gene products have been identified as highly protective when induced before and, in some cases after the start of ischemic injury. In general, these components are involved in scavenging of ROS or detoxifying enzymes capable of removing ROS [36]. Among others, GSS is well known to detoxify hydrogen peroxide as well as peroxynitrate by increasing intracellular glutathione levels [38,39]. Recent studies examined the therapeutic potential of GSS, a compound of particular clinical interest in humans which is able to react spontaneously with nearly every oxidants formed during inflammation and reperfusion [40]. One of the first antioxidants investigated was the use of exogenous catalases as well as SOD [41]. However, administration of SOD resulted in only partial protection due to its poor bioavailability. Up-regulation of SODs is known to scavenge ROS and prevent the formation of peroxynitrites [42,43].

In the course of this study, we found significantly elevated SOD2 levels in liver tissue in BD after perfusion and after CIT indicating that BD might induce protective effects in the liver prior to transplantation. Interestingly we could not detect these changes in kidney tissue of BD, but in kidneys of LD SOD2 as well as OXSR1 were significantly elevated. Taking in consideration the better results of LD kidney transplantation compared to BD kidney transplantation, the over-expression of these genes might be one of the reasons for less IRI and DGF. Another gene that protects cells against oxidative stress induced cell damage is GPX3 [17,18]; however, in contrast in our study GPX3 was only over-expressed after perfusion in LD livers, but not after CIT, suggesting that this gene can be neglected in the pathophysiological pathway of occurrence of apoptosis and graft survival in a transplantation setting. OXSR1 is known to be involved in cytoskeleton rearrangements and reacts on osmotic stress and controls whether cells proliferate or die by apoptosis [44]. Interestingly, OXSR1 levels were significantly higher in LD compared to BD after CIT in kidney tissue. However, in a LD setting, usually there is less CIT and therefore, the protective effects of these genes might not be as important as in a BD setting. Other genes involved in organ protection belong to the HSP 70 family which is capable of protecting cells from lethal heat and other insults [45] and of preventing protein aggregation and facilitates the refolding of denaturated proteins. Up-regulating HSP 70 as a pre-conditioning strategy has been shown to be cytoprotective in a number of organs including the kidney and heart [5,46]. HSP 70 has immune regulation properties with both pro- and anti-inflammatory effects [47] and large clinical studies focusing on cardiac ischemia have provided inconsistent reports of protective [48,49] or detrimental effects [50] on the tissue. Induction of HSP 70.2 increased survival and protected against IRI in the liver [51] as well as in the kidney [5]. In summary, the functions of HSP 70 are supposed to depend on its location with intracellular HSP 70 attenuating inflammatory cascades, while HSP 70 released into the extracellular matrix produces immune-regulatory effects [52,53]. The results of our study showed significantly higher levels of HSP 70.2 expression in heart tissue after perfusion and CIT in BD whereas in kidney tissue there was only a significant difference between prior to perfusion and after CIT in LD organs and remained stable in BD organs, indicating the protective effects of HSP 70.2 in the LD setting after CIT; these results are not in accordance with a recent study of van Dullemen et al. [5] who showed enhanced expression of genes of the HSP family in the kidney what might to be due to the fact that they used a shorter period of BD as compared to our study. However, they stated in their study that an increase of HSP 70 expression appears to be insufficient to counteract the detrimental effects of BD to the donor kidney [5]; therefore, taking in consideration the results of our study, the duration of BD especially in our large animal model, might negatively impact on the expression of genes of the HSP 70 family indicating that BD time should be kept as short as possible prior to organ retrieval but pharmacological intervention enhancing HSP expression prior to organ donation might be a valuable procedure to increase organ quality of BD kidneys [5].

PPAR-alpha is crucial in lipid metabolism and regulation of inflammation [15]. PPAR-alpha has been most extensively studied in the regulation of genes involved in glucose and lipid metabolism [54]. However, it is also expressed by macrophages, T-cells, dendritic cells, endothelial cells and other cell types, that impact on inflammation and immunity [55-57]. PPAR-alpha ligands are known to inhibit interleukin-2 (IL-2), tumor necrosis factor alpha and interferon-gamma production by activated T-cells [58] and therefore might be involved in acute rejection [15]. In our study PPAR-alpha levels did not change significantly in any organ investigated over time.

If the protective mechanisms described above are altered, apoptosis may occur, leading to graft dysfunction and patient mortality. Therefore we additionally investigated pro-apoptotic BAX [8] and anti-apoptotic genes like BCL2L1 [9] or NFKB [7] which is known to play a key role in initiation of inflammation and rejection as well as occurrence of apoptosis in organ transplantation [10]. The ratio between BAX and BCL2L1 gene expression, both mitochondria-associated proteins defines the susceptibility of cells towards apoptotic stimuli [11]. BCL2L1 was significantly higher after 4 hours of CIT in heart tissue of BD donors. BCL2L1 levels of LD was significantly higher at all time points observed in kidney tissue whereas no significant changes could be found in BCL2L1 levels in liver tissue in between LD and BD over the whole observation period. This indicates through the up-regulation of this anti-apoptotic gene that BD seems to have protective effects in terms of inactivation of the apoptotic pathway in heart tissue, whereas no influence could be detected in BD liver tissue. The same results were found for the pro-apoptotic gene BAX that was up-regulated after CIT in heart tissue of BD but not in liver as well as kidney tissue. Similar results were observed for NFKB which, however, was not up-regulated after CIT in heart tissue as well as at all time points observed in liver tissue regarding BD. The balance between the anti-apoptotic gene expression and the pro-apoptotic gene expression in these cases might be caused by the up-regulation of genes protecting from oxidative stress induced cell damage as mentioned above. These findings are not in accordance with a study of Van der Hoeven et al. [6] who showed that mRNA levels of several anti-apoptotic proteins were up-regulated in BD rat livers including the BCL2 family; However, the main difference in between these two studies is, that in our study, we used a large animal model and a longer period of BD. Moreover, our study focused more detailed on the expression of oxidative stress induced gene expression and therefore these two studies seem not to be adequately comparable but when being taken together might help to get better insights in the pathophysiological pathways occurring during BD what poses the possibility to design new studies in order to find strategies to improve organ quality after BD and consequently improve transplantation outcome. Interestingly, NFKB was up-regulated in LD kidney tissue after CIT. In order to confirm the results from the panel of genes we investigated, immunohistochemical staining for apoptotic and nitrosative stress induced cell damage were performed to show that the cascades mentioned above helped to prevent cell damage or not.

For histological evaluation of apoptotic cell death, an antibody against activated Caspase 3 was chosen in this study due to its reliability and because it is involved in the final execution phase of apoptosis [59]. Due to the fact that in our laboratory immunohistochemistry for activated Caspase 3 is well established and this staining method is recommended for detection of the onset of the irreversible pathway of apoptosis we decided to use this staining method in this study [60]. In all organs investigated in this study we found elevated levels of activated Caspase 3 positive cells in BD as compared to LD, especially in the liver being significantly elevated even prior to organ retrieval indicating that BD induces apoptotic cell death due to the hemodynamic instability including short hypertensive phases at the onset of BD followed by a decline of blood pressure to normal tension or hypotension, what could cause poor organ perfusion and thereby inadequate oxygen supply despite of intensive donor care during 10 hours of BD. In all other organs, we could observe a significant increase in apoptotic cells after the different CIT. In liver and kidney tissue apoptotic cell death was less pronounced in LD compared to BD organs after CIT, whereas in heart tissue no such difference was found. We are aware of the fact that LD of heart is only a hypothetical scenario, but from our point of view these control organs taken from LD helped to get better insights in the pathophysiological cascades during BD and CIT impacting on organ quality and graft survival. These findings indicate that although organs from BD donors do not show more apoptosis before and after perfusion, organs from BD animals seem to be more vulnerable to CIT. This is interesting due to the fact that from this point of view, the donor source does not seem to influence the organ quality in terms of apoptotic cells, but CIT. These findings are in accordance with findings of another study of our group where we could show that the levels of high energy phosphates as an indicator of organ quality are independent of the donor type but dependent on CIT [22].

The second marker, which was chosen for immunohistochemical evaluation of LD and BD tissue was nitrotyrosine, a specific marker for nitrosative stress [61] which is known to negatively influence organ function. Except for heart tissue we found significantly different amounts of nitrotyrosine positive cells after BD at the time prior to organ retrieval indicating that BD induces nitrosative stress mainly supposed through less oxygen supply due to hemodynamic disturbances caused by the “autonomic storm” induced by BD. Nitrosative stress seems to be the highest in liver tissue of BD as compared to LD. However, CIT did not significantly influence nitrotyrosine expression in kidney tissue whereas in heart and liver tissue there was also a significant elevation of nitrotyrosine positive cells when comparing LD and BD. Esposito et al. recently reported to reduce nitrosative stress in IRI after organ transplantation by administration of glutamine [61] what seems to be a promising method to reduce nitrosative stress induced cell damage as well as lipidperoxidation when regarding the results of our study, especially in BD organs when a certain CIT has to be considered. In kidney and liver tissue of BD organs, activated Caspase 3 as well as nitrotyrosine positive cells were mainly found next to blood vessels, indicating the vulnerability of the vascular endothelium to oxidative and nitrosative stress. In kidney tissue, especially tubular tissue seemed to be more susceptible to occurrence of apoptosis after CIT.

Among other processes occurring during BD, activated neutrophils secrete enzymes such as MPO and liberate more ROS [62]. MPO is known as an inflammation marker. However, we did not observe significant differences in MPO concentrations in the serum of LD and BD pigs indicating that in our study, the MPO concentrations were not elevated through the induction of BD and therefore we can exclude its negative impact on organ quality in the LD and BD setting of our study. Moreover, ROS may lead to severe injury to the cell membrane by lipid peroxidation which may generate reactive carbonyl compounds such as MDA the most abundant aldehyde resulting from lipid peroxidation [63,64]. However, we could not detect any differences in MDA concentrations in the serum of LD and BD pigs.

## Conclusion

The results of this study are consistent and show that pro-apoptotic genes as well as genes supposed to be protective in terms of reduction of oxidative stress induced cell damage are differentially regulated during BD compared to LD depending on the type of organ. Low levels of oxidative stress are postulated to be responsible for pre-conditioning effects such as elevation of GSS, SOD as well as HSP 70.2 levels [65]. Despite the up-regulation of these protective genes, at the end of CIT, all BD organs showed significantly more apoptotic cells as compared to LD. However, it can be speculated that, if the pre-conditioning of the organs during BD would be missing, the final outcome after CIT would even be more detrimental, due to the lack of the protective effects of the panel of genes analyzed. The pathophysiological pathways finally resulting in more apoptotic and nitrotyrosine positive cells after CIT in BD seem to be different dependent on the type of organ. One limitation of this study is that it was not performed in a human setting, but since a study like this would not be possible using human donor organs, a large reproducible animal model is the best alternative. To our knowledge this is the first large animal study focusing on this panel of genes in a BD as well as LD setting comparing these results with immunohistochemistry. We are convinced that we could gain more detailed insights in the pathophysiological pathways occurring during BD and, moreover, during CIT focusing on the different types of organs. Further studies will be necessary to show the detailed effects of different substances, which might be used for donor pre-conditioning in BD helping to improve transplantation outcome.

## Abbreviations

BD: Brain death; BAX: BCl2 associated X protein; BCL2L1: B-cell lymphoma 2 like 1; CIT: Cold ischemic time; DGF: Delayed Graft Function; GPX3: Gluthatione peroxidase 3; GSS: Gluthatione synthetase; HPRT1: Hypoxanthine phosphoribosyltransferase; HSP: 70.2 Heat shock protein 70.2; IRI: Ischemia reperfusion injury; LD: Living donation; MDA: Malondialdehyde; MPO: Myeloperoxidase; NFKB: Nuclear factor of kappa light polypeptide gene enhancer in b-cells; OXSR1: Oxidative stress responsive 1; PCR: Polymerase chain reaction; PPARalpha: Peroxisome proliferators-activated receptor-alpha; RNA: Ribonucleic acid; SOD: Superoxide dismutase.

## Competing interests

The authors declare that they have no competing interest.

## Authors’ contributions

VS, PS and MS planned the experiments and wrote the article, PS, MS, AP and AB performed the animal experiments VS performed the statistical analysis. BL performed the immunohistological analysis and the PCR analysis. ES and ND planned and performed the PCR analysis and designed the primer therefore. TSH performed EEG readings and BD diagnosis. IW performed anaesthesia on the animals. SZ performed routine laboratory analysis and MDA and MPO measurement. FI planned the experiments and reviewed the article. All authors read and approved the final manuscript.
